# Starvation-induced suppression of DAZAP1 by miR-10b integrates splicing control into TSC2-regulated oncogenic autophagy in esophageal squamous cell carcinoma

**DOI:** 10.7150/thno.43046

**Published:** 2020-04-06

**Authors:** Yunsong Chen, Yan Lu, Yanli Ren, Jupeng Yuan, Nasha Zhang, Hannah Kimball, Liqing Zhou, Ming Yang

**Affiliations:** 1 Shandong Provincial Key Laboratory of Radiation Oncology, Cancer Research Center, Shandong Cancer Hospital and Institute, Shandong First Medical University and Shandong Academy of Medical Sciences, Jinan, Shandong Province, 250117, China; 2 Department of Medical Oncology, Dana-Farber Cancer Institute, Harvard Medical School, Boston, MA, 02215, USA; 3 Department of Radiation Oncology, Huaian No. 2 Hospital, Huaian, Jiangsu Province, 223002, China

**Keywords:** autophagy, selective splicing, miR-10b, DAZAP1, TSC2, esophageal squamous cell carcinoma

## Abstract

Esophageal squamous cell carcinoma (ESCC) accounts for about 90% of all incident esophageal cancers, with a 5-year survival rate of < 20%. Autophagy is of particular importance in cancers; however, the detailed regulatory mechanisms of oncogenic autophagy in ESCC have not been fully elucidated. In the present study, we address how splicing control of *TSC2* is involved in mTOR-regulated oncogenic autophagy.

**Methods:** Alternative splicing events controlled by DAZAP1 in ESCC cells were identified via RNAseq. Differential phosphorylation of short or long TSC2 splicing variants by AKT and their impacts on mTOR signaling were also examined.

**Results:** We found that starvation-induced miR-10b could enhance autophagy via silencing DAZAP1, a key regulator of pre-mRNA alternative splicing. Intriguingly, we observed a large number of significantly changed alternative splicing events, especially exon skipping, upon RNAi of DAZAP1. *TSC2* was verified as one of the crucial target genes of DAZAP1. Silencing of DAZAP1 led to the exclusion of *TSC2* exon 26 (from Leu947 to Arg988), producing a short TSC2 isoform. The short TSC2 isoform cannot be phosphorylated at Ser981 by AKT, which resulted in continuous activation of TSC2 in ESCC. The active TSC2 inhibited mTOR via RHEB, leading to continually stimulated oncogenic autophagy of ESCC cells.

**Conclusions:** Our data revealed an important physiological function of tumor suppressor DAZAP1 in autophagy regulation and highlighted the potential of controlling mRNA alternative splicing as an effective therapeutic application for cancers.

## Introduction

Autophagy is an evolutionarily conserved catabolic process that degrades and recycles damaged proteins and organelles as well as dangerous cytosolic entities (e.g., invading pathogens). Upon sequestration, these components are enclosed within double-membraned vesicles (autophagosomes) and delivered to lysosomes for degradation 1-3. After the contents of autolysosomes fuse with lysosomes, different enzymes cleave the cargo into products such as amino acids, nucleic acids, sugars, and fatty acids that can be reutilized by the cell 4-6. Nutrient starvation, hypoxia, oxidative stress, and infection can induce autophagy to allow for adaptation and cell survival 7,8. Autophagy is active at a low basal level to sustain homeostasis of cells by eliminating damaged organelles and protein aggregates in normal tissues 1-3. The role of autophagy in cancers is of particular importance since the increased autophagic flux prevents malignant transformation of normal cells, whereas enables tumor cell survival and growth once cancers are established 9,10.

As the sixth leading cause of cancer death worldwide, esophageal cancer is a complex disease with many causes, which differ by histologic types and the populations 11-13. Esophageal squamous cell carcinoma (ESCC) accounts for about 90% of all incident esophageal cancers each year, with < 20% of a 5-year survival rate 11-13. Previous studies indicated that autophagy acts as tumor-promoting factor in ESCC cells, permitting the expansion and maintenance of cancer stem cells with enhanced malignant potential as well as metastasis-associated phenotypes 14,15. For example, high CD44 expression is associated with enhanced malignant potential in ESCC. Autophagy facilitates epithelial-mesenchymal transition of transformed esophageal keratinocytes with high CD44 expression via modulation of redox homeostasis and Parkin-dependent mitochondrial clearance 16. Additionally, esophageal cancer stem cells represent a subpopulation of ESCC that exhibit the capacity for tumor initiation and progression. Autophagy maintains the stem-like properties of OV6-positive cells by stabilizing ATG7-dependent β-catenin, which promotes the progression of ESCC 17.

Recent evidence demonstrated that miRNAs play a vital role in the regulation of various cellular processes in cancer development including autophagy 18, though the importance within ESCC is not fully understood. For instance, miR-634 has been found to impair autophagic degradation, activate the mitochondrial apoptosis pathway and enhance chemotherapy-induced cytotoxicity of ESCC 19. Nyhan et al. reported that miR-193b promotes autophagy and nonapoptotic cell death in ESCC cells 20. We previously found that miR-638 promotes autophagy and malignant phenotypes of ESCC cells via directly suppressing *DACT3* 21. Thus, multiple miRNAs are involved in the regulation of autophagy; however, the molecular mechanistic details of how these miRNAs regulate autophagy and their impacts on ESCC development have not been fully characterized.

MiR-10b is overexpressed in multiple cancers such as ESCC, breast cancer, glioma, hepatocellular carcinoma and acute myeloid leukemia 22-28. Importantly, miR-10b was the first miRNA which was identified to affect invasion and metastasis of human cancers 24. Tian et al. found that overexpression of miR-10b in ESCC KYSE140 cells increased cell motility and invasiveness, whereas inhibition of miR-10b in EC9706 cells reduced cell invasiveness 23. They identified *KLF4* (*Krüppel-like factor 4*) as a direct target of miR-10b and speculated that the oncogenic function of miR-10b may be partially mediated by KLF4 in ESCC 23. mTOR senses deficient nutrient and energy status to induce autophagy of cells in response to environmental changes, such as starvation. Surprisingly, activation of mTOR caused a broad reduction in miRNAs due to Drosha degradation. mTOR activation increased expression of Mdm2, which is hereby identified as the necessary and sufficient ubiquitin E3 ligase for Drosha, a key miRNA biogenesis enzyme in processing of primary miRNA to precursor miRNA 29,30. Consistently, elevated expression of miR-10b was observed in lung cancer cells after starvation 31, indicating that miR-10b might be involved in starvation-induced autophagy. Considering a single miRNA generally has multiple targets involved in different biological events, we hypothesized that miR-10b might be involved in starvation-induced autophagy of ESCC.

In this study, we identified miR-10b functioning as a key regulator of starvation-induced autophagy of ESCC cells. Suppression of *DAZAP1* by miR-10b could lead to alternative splicing of *TSC2* exons, compromising mTOR activities to enhance oncogenic autophagy.

## Results

### MiR-10b promotes starvation-induced autophagy in ESCC cells

**Figure 1:**
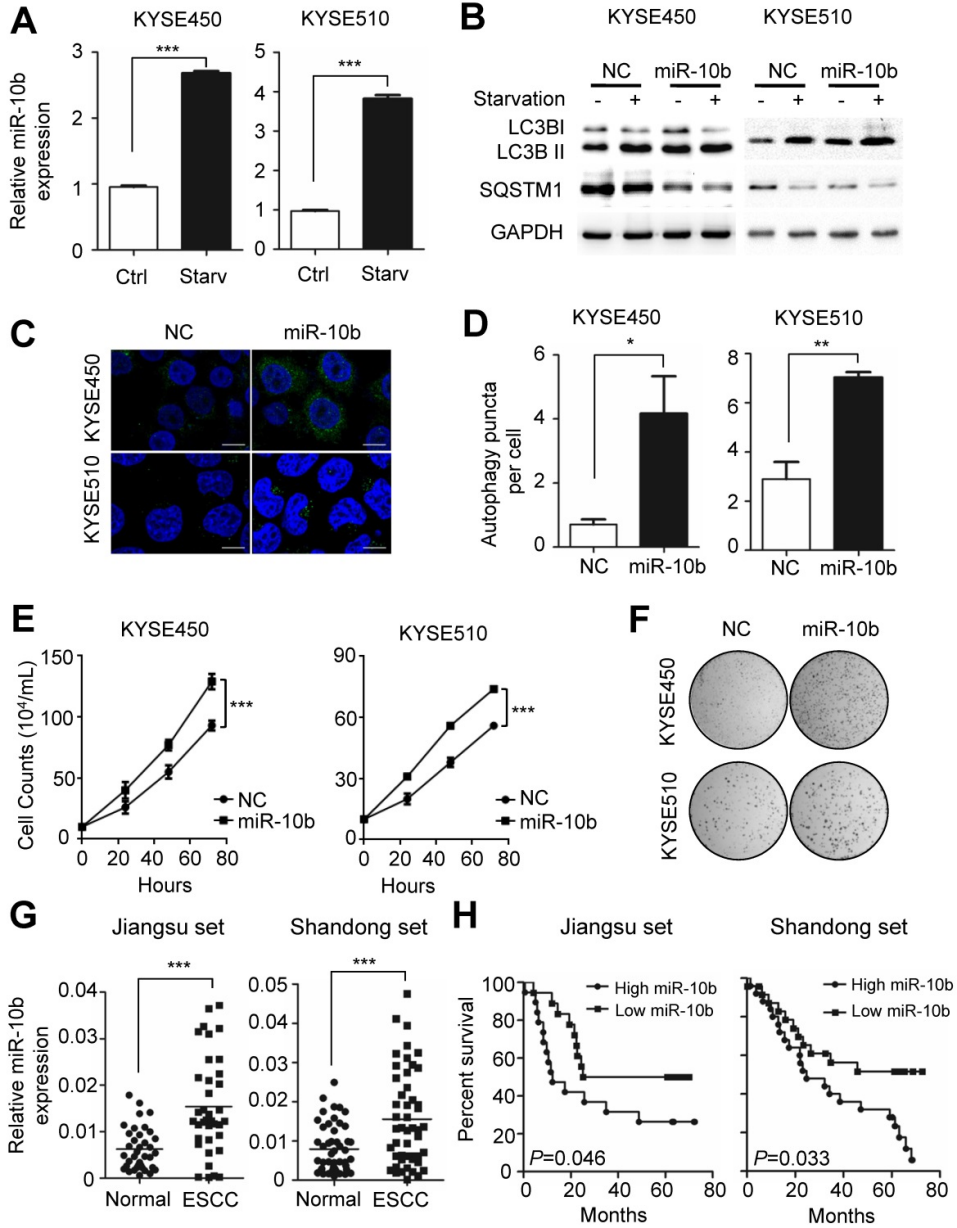
** Nutrient deprivation-induced miR-10b promotes autophagy and functions as an oncogene in ESCC. (A)** Nutrient deprivation significantly induced the miR-10b expression in ESCC KYSE450 and KYSE510 cells. **(B)** Over-expressed miR-10b increased starvation-induced conversion of LC3-I to LC3-II as well as starvation-induced SQSTM1 degradation in KYSE450 and KYSE510 cells. **(C, D)** miR-10b enhanced accumulation of autophagosomes which were visualized via the LC3B-II immunofluorescence in KYSE450 and KYSE510 cells. Scale bars, 10 μm. The number of LC3 puncta in cells of each group was calculated from 3 random fields, and at least 30 cells were chosen. **(E, F)** miR-10b significantly promotes proliferation of KYSE450 and KYSE510 cells.** (G)** There was elevated miR-10b expression in ESCC tissues compared to normal tissues of cases from Shandong set or the Jiangsu set. **(H)** Increased miR-10b expression was significantly associated with poor survival of ESCC patients. All results of the mean of triplicate assays with standard deviation are presented. The difference between two groups was calculated using Student's *t* test (assuming Gaussian distributions) or Wilcoxon Signed Rank Test (not assuming Gaussian distributions). ^*^P < 0.05, ^**^P < 0.01, ^***^P < 0.001.

Autophagy plays an essential role in sustaining ESCC growth and progression. Previously, miR-10b has been shown to promote migration and invasion, via KLF4, in human esophageal cancer cell lines 23. From these results, we speculated the potential involvement of miR-10b in the regulation of autophagy, thus impacting ESCC progression. We examined whether endogenous miR-10b levels were responsive to the starvation-inducing stimuli in ESCC cells. A significant increase in miR-10b expression levels was observed in both KYSE450 and KYSE510 cell lines after cells were starved for 4h via EBSS (Earle's Balanced Salt Solution) treatment (both *P*<0.001) (Figure 1A). Considering activation of mTOR could suppress Drosha expression and, thus, miRNAs expression 29,30, we examined miR-10b levels in KYSE450 and KYSE510 cells treated with a mTOR activator, MHY1485. Strikingly decreased miR-10b expression was observed in ESCC cells after MHY1485 treatment (Figure S1A), indicating that starvation induced nutrient-deprivation may lead to inhibited mTOR, elevated Drosha expression and increased miR-10b biogenesis. We then tested if miR-10b could impact starvation-induced autophagy in KYSE450 and KYSE510 cells (Figure 1B-1D and Figure S1B-S1C). We found that ectopic expression of miR-10b promoted the conversion of MAP1LC3B-I (microtubule associated protein 1 light chain 3 beta, also known as LC3B-I) to LC3B-II, a hallmark of autophagosome formation (Figure 1B). Additionally, miR-10b accelerated degradation of the autophagy receptor, SQSTM1, after starvation (Figure 1B) and significantly enhanced the accumulation of autophagosomes, which were visualized via LC3B-II immunofluorescence staining (both *P*<0.05) (Figure 1C and 1D). These results demonstrated that miR-10b's involvement in controlling starvation-induced autophagy.

We further evaluated the role of miR-10b-related autophagic flux in ESCC development. As shown in Figure 1E, miR-10b could significantly promote proliferation of both KYSE450 and KYSE510 cells (both *P*<0.001). Similarly, miR-10b was able to stimulate colony formation of KYSE450 and KYSE510 (Figure 1F), indicating that miR-10b contributes to oncogenic growth of ESCC cells. In support of the oncogenic nature of miR-10b, the association of an evidently increased miR-10b expression with ESCC tissues was observed, comparing to normal tissues of cohorts from Jiangsu set or Shandong set (both *P*<0.001) (Figure 1G and Figure S1D). The elevated miR-10b expression was also significantly associated with poor prognosis of ESCC patients from both sets (both log-rank *P*<0.05) (Figure 1H).

### Identification of DAZAP1 as a direct target of miR-10b in ESCC

**Figure 2:**
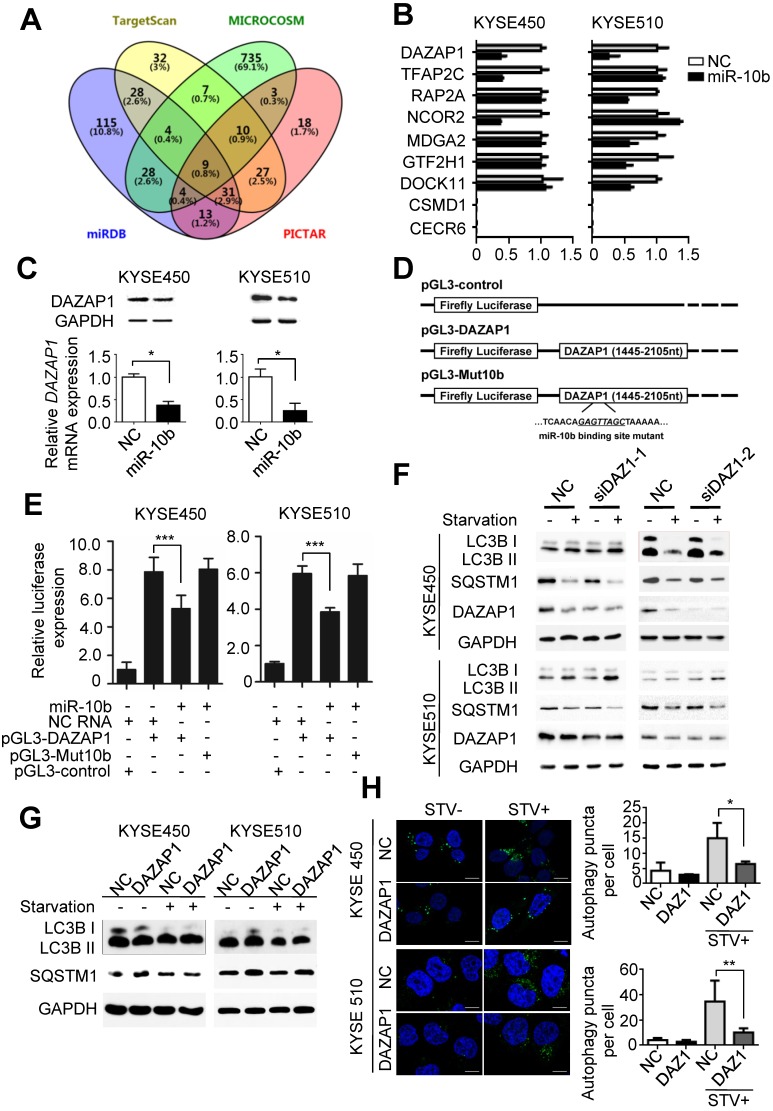
** MiR-10b promotes autophagy by silencing *DAZAP1* expression in ESCC. (A)** Venn diagram of potential candidate target genes of miR-10b by integrating the results of the algorithms TargetScan, PICTAR, Micro-RNA and MiRDB. **(B)** qRT-PCR validation of the nine potential target genes of miR-10b in KYSE450 and KYSE510 cells transfected with either miR-10b mimics or NC RNA. **(C)** miR-10b could significantly inhibit DAZAP1 protein and mRNA expression in ESCC celllines. **(D)** Schematic constructions of pGL3-DAZAP1 and pGL3-Mut10b. **(E)** pGL3-DAZAP1 and pGL3-Mut10b were co-transfected into KYSE450 and KYSE510 cells with miR-10b mimics or NC RNA. Luciferase activity was detected at 48h after transfection and normalized relative to the Renilla luciferase expression. Inhibition effects of miR-10b mimics on pGL3-DAZAP1 or pGL3-Mut10b were showed. **(F, G)** Immunoblot results of extracts from non-starved or starved KYSE450 and KYSE510 cells. Silencing *DAZAP1* expression (siDAZ1-1 and siDAZ1-2) increased starvation-induced conversion of LC3B-I to LC3B-II and accelerated rapamycin-induced SQSTM1 degradation in both ESCC celllines. Over-expressed DAZAP1 suppressed conversion of LC3B-I to LC3B-II and down-regulation of SQSTM1 in KYSE450 and KYSE510 cells. **(H)** DAZAP1 inhibited starvation-induced GFP-LC3 LC3B^+^ autophagosomes formation in both ESCC celllines. The number of LC3 punctae in cells of each group was calculated from 3 random fields, and at least 30 cells were chosen. Autophagy was assessed under non-starved (STV-) or starved (STV+) conditions. The difference between two groups was calculated using Student's *t* test (assuming Gaussian distributions) or Wilcoxon Signed Rank Test (not assuming Gaussian distributions). All results of the mean of triplicate assays with standard deviation are presented.^ *^P < 0.05; ^**^P < 0.01; ^***^P < 0.001.

To further investigate the mechanisms of miR-10b involved in promoting oncogenic autophagy, its potential candidate target genes were predicted by integrating results from different algorithms including TargetScan, PICTAR, Micro-RNA and MiRDB (Figure 2A). Nine overlapped candidate target genes (*DAZAP1*, *TFAP2C*,* RAP2A*, *NCOR2*, *MDGA2*, *GTF2H1*, *DOCK11*, *CSMD1* and* CECR6*) were identified through Venny 2.1.0 analyses (Figure 2A). Then, we examined the impact of miR-10b on the expression level of each candidate gene in ESCC cell lines. After transfection with miR-10b mimics or NC RNA as the negative control, the endogenous expression levels of these nine candidate genes in KYSE450 and KYSE510 cells were determined (Figure 2B). We found that the expression levels of *TFAP2C*,* RAP2A*, *NCOR2*, *MDGA2*, *GTF2H1*, and *DOCK11* were down-regulated in one of the two ESCC cell lines, while the expression of *DAZAP1* was inhibited by the ectopic miR-10b in both KYSE450 and KYSE510 cell lines (Figure 2B). Additionally, miR-10b significantly inhibited DAZAP1 protein expression in both ESCC cell lines (Figure 2C and Figure S2A). From these results, we focused on the investigation of the role of DAZAP1 in ESCC. Dual luciferase reporter gene assays were conducted to examine the potential direct interaction between miR-10b and the *DAZAP1* 3'UTR. We first subcloned a 661bp human *DAZAP1* 3'UTR sequence linked to the firefly luciferase gene (pGL3-DAZAP1) (Figure 2D). Point substitutions were introduced to pGL3-DAZAP1 to disrupt the binding site of miR-10b in the 3'UTR of the construct (pGL3-Mut10b) (Figure 2D). KYSE450 and KYSE510 cells were co-transfected with pGL3-DAZAP1 and miR-10b mimics or NC RNA. We found a 32.6% or 35.2% decrease in luciferase activity in the miR-10b transfected group compared to the NC RNA group in KYSE450 or KYSE510 cells (both *P*<0.001) (Figure 2E). However, no significant reduction of luciferase activities caused by miR-10b was observed in ESCC cells that were co-transfected with pGL3-Mut10b and miR-10b mimics or NC RNA (both *P*>0.05) (Figure 2E).

To verify whether DAZAP1 is involved in miR-10b-related autophagic flux in ESCC, we examined the impact of DAZAP1 on the starvation-induced autophagy of KYSE450 and KYSE510 cells (Figure 2F-2H). After silencing of DAZAP1 with siRNAs (siDAZ1-1 and siDAZ1-2), we found an increased conversion of LC3B-I to LC3B-II and a significantly decreased SQSTM1 expression after starvation (Figure 2F and Figure S2B-2C). In contrast, ectopic expression of DAZAP1 could suppress the conversion of LC3B-I to LC3B-II and the up-regulation of SQSTM1 (Figure 2G and Figure S2D-2E). The exogenous over-expression of DAZAP1 significantly reduced starvation-induced accumulation of autophagosomes in KYSE450 and KYSE510 cells (both *P*<0.05) (Figure 2H). Ectopic expression of DAZAP1 could rescue starvation-induced autophagy enhanced by miR-10b in KYSE450 and KYSE510 cells (Figure S3), demonstrating that DAZAP1 is a key downstream target of miR-10b in regulating starvation-induced autophagy.

### DAZAP1 acts as a tumor suppressor in ESCC

**Figure 3:**
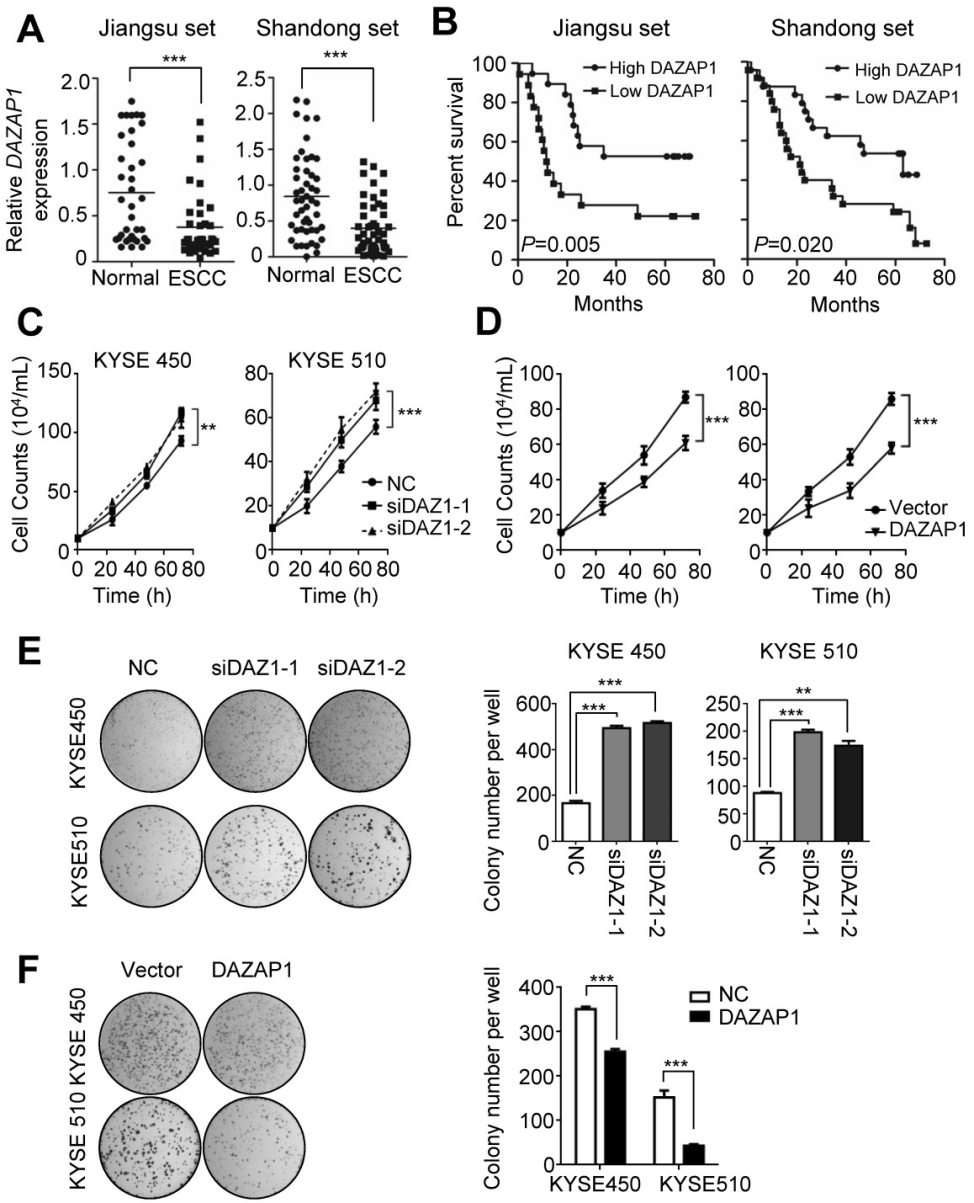
** DAZAP1 inhibits cell proliferation of ESCC cells. (A)** Relative *DAZAP1* expression in 86 pairs of ESCC tissues and normal esophageal tissues (Jiangsu set and Shandong set). Significantly down-regulated *DAZAP1* expression was observed in ESCC tissues compared with normal esophageal samples in both patient sets. **(B)** ESCC patients with high *DAZAP1* expression exhibited significantly prolonged survival.** (C, D)** Silencing *DAZAP1* expression (siDAZ1-1 and siDAZ1-2) promoted cell proliferation. However, ectopic *DAZAP1* expression inhibited KYSE450 and KYSE510 cell growth. Cell numbers were counted at 24h, 48h and 72h after transfection. **(E, F)** Colony formation assays. On the 14th day after transfection of* DAZAP1* siRNAs or expression constructs, colony number in each well was counted. The difference between two groups was calculated using Student's *t* test (assuming Gaussian distributions) or Wilcoxon Signed Rank Test (not assuming Gaussian distributions). All results of the mean of triplicate assays with standard deviation are presented. ^**^P < 0.01; ^***^P < 0.001.

The highly conserved RNA binding protein DAZAP1 was originally identified as a binding partner of DAZ (deleted in azoospermia) 32,33 and is involved in mammalian development and spermatogenesis 34. To explore the potential role of *DAZAP1* in ESCC development, we examined *DAZAP1* expression in 86 pairs of ESCC tissues and normal esophageal tissues (Jiangsu set and Shandong set). There was significant down-regulated *DAZAP1* expression in ESCC tissues compared to normal esophageal samples in both patient sets (both *P* < 0.001) (Figure 3A and Figure S4A-4B). ESCC patients with a relatively high *DAZAP1* expression exhibited significantly longer survival time compared to cases with a relatively low expression (both log-rank *P*<0.05) (Figure 3B). Additionally, we observed an evident negative expression correlation between *DAZAP1* and miR-10b in tissue samples from both Jiangsu set and Shandong set (Figure S4C-4F).

The tumor suppressor function of *DAZAP1* was further determined in ESCC cell lines (Figure 3C-3F). DAZAP1 significantly suppressed the viability of KYSE450 and KYSE510 cells (Figure 3C and 3D). Either siDAZ1-1 or siDAZ1-2 could comparably stimulate proliferation of KYSE450 cells, 72h after transfection (both *P*<0.01). Consistently, we observed a significantly enhanced viability of KYSE510 cells, 72h after *DAZAP1* siRNA delivery (both *P*<0.001). In contrast, the ectopic DAZAP1 expression could inhibit cell proliferation of KYSE450 and KYSE510 cells (Figure 3D). Congruent with cell viability assays, *DAZAP1* siRNA accelerated colony formation of KYSE450 and KYSE510 cells (all *P*<0.01) (Figure 3E), while the over-expression of DAZAP1 significantly suppressed colony formation of ESCC cells (both *P*<0.001) (Figure 3F).

### MiR-10b promotes migration and invasion of ESCC cells via targeting DAZAP1

**Figure 4:**
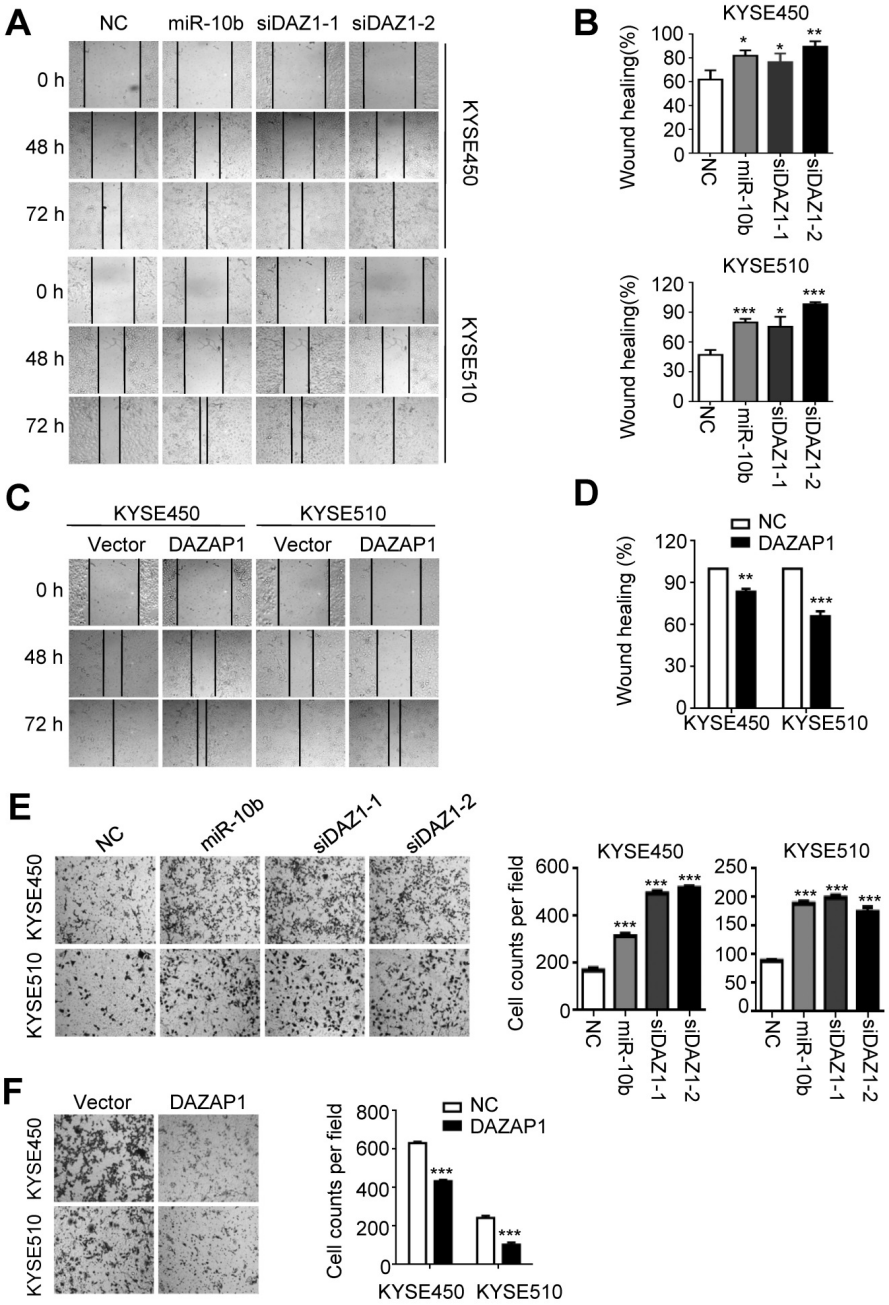
** MiR-10b increases migration and invasion abilities of ESCC cells via targeting DAZAP1. (A,B)** miR-10b and DAZAP1 RNAi (siDAZ1-1 and siDAZ1-2) promoted wound-healing of KYSE450 and KYSE510 cells. Wound-healing area in both celllines is presented as a histogram. **(C, D)** Over-expressed DAZAP1 evidently inhibited wound-healing. **(E, F)** miR-10b and silencing DAZAP1 promoted invasion ability of KYSE450 and KYSE510 cells. However, ectopic *DAZAP1* expression suppressed invasion ability of both ESCC celllines. Cells on the lower surface of the chamber were stained by crystal violet at 48 h after small RNA transfection. Cell counts data are presented as a histogram. The difference between two groups was calculated using Student's *t* test (assuming Gaussian distributions) or Wilcoxon Signed Rank Test (not assuming Gaussian distributions). All results of the mean of triplicate assays with standard deviation are presented.^ *^P < 0.05; ^**^P < 0.01; ^***^P < 0.001. (siDAZ1-1 and siDAZ1-2)

Next, we investigated how miR-10b and *DAZAP1* impact the capability of migration and invasion of ESCC cells. As shown in Figure 4A and 4B, miR-10b mimics and *DAZAP1* siRNAs significantly enhanced the motility of both KYSE450 and KYSE510 cells compared to control cells transfected with NC RNA (at 72h after transfection, all *P*<0.05). Conversely, we observed the impaired motility capability of KYSE450 and KYSE510 cells after elevated expression of DAZAP1 (at 72h after transfection, both *P*<0.001) (Figure 4C and 4D). Consistent with wound-healing assays, miR-10b, siDAZ1-1 or siDAZ1-2 could significantly accelerate migration of ESCC cells (all *P*<0.001) (Figure 4E). In contrast, the ectopic DAZAP1 suppressed invasion ability of ESCC cells (all *P*<0.001) (Figure 4F). Our findings suggest that *DAZAP1* acts as a crucial tumor suppressor in ESCC pathogenesis.

### DAZAP1 regulates alternative splicing of TSC2 mRNA

**Figure 5:**
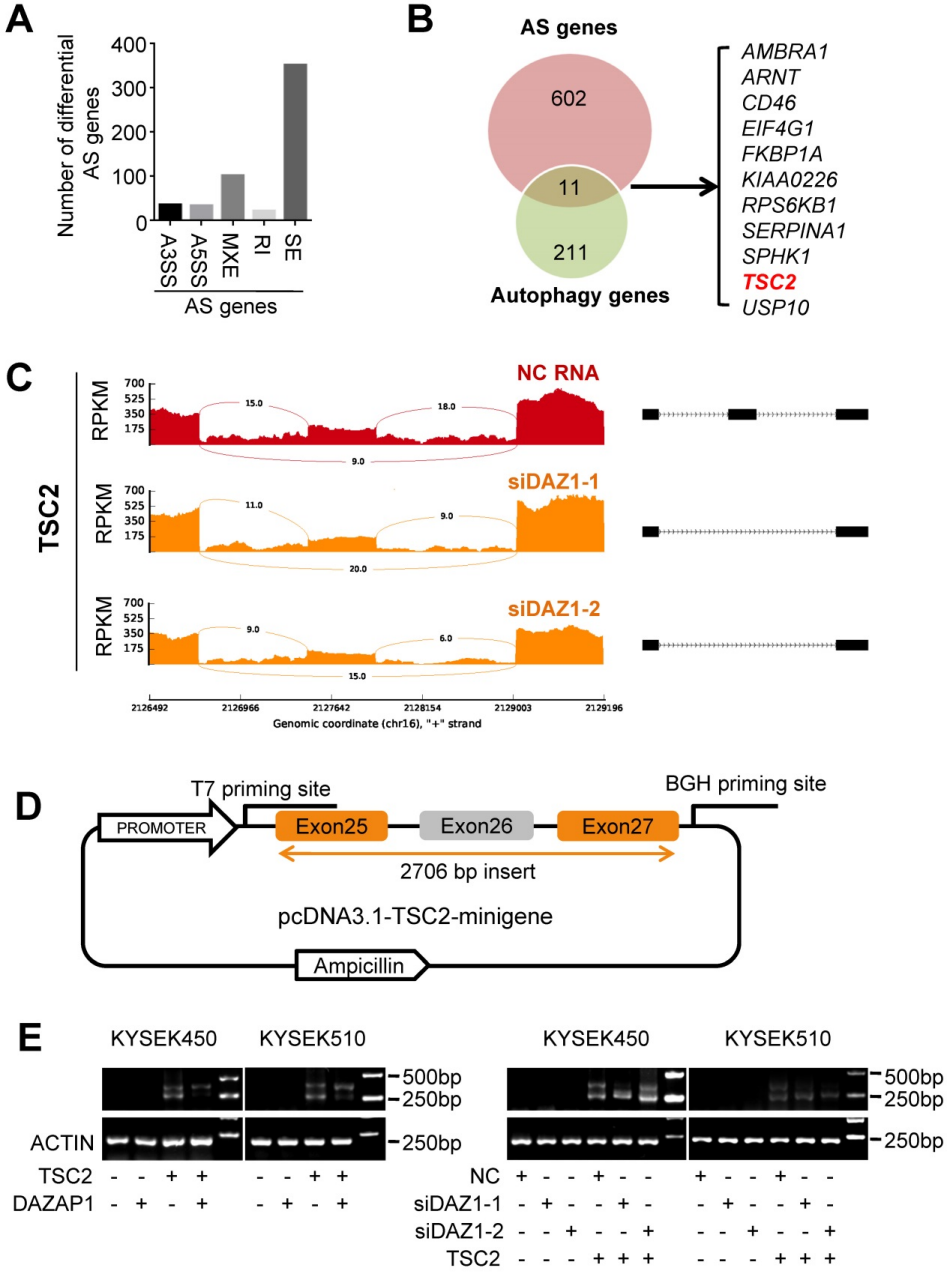
** DAZAP1 regulates alternative splicing of *TSC2* mRNA. (A)** RNAseq of KYSE510 cells transfected with siRNAs (siDAZ1-1 and siDAZ1-2) or NC RNA was performed to identify endogenous splicing events controlled by DAZAP1. MATS analyses indicate that dysregulated DAZAP1 led to six hundred and thirteen alternative splicing events, including exon skipping (SE,* n* = 352), intron retention (RI, *n* = 22), alternative 5' splice site (A5SS, *n* = 34), alternative 3' splice site (A3SS, *n* = 36), and mutually exclusive exon (MXE, *n* = 102). **(B)** Venn diagram of the overlapped genes between the alternative splicing (AS) genes and autophagy genes. **(C)** Decreased inclusion of *TSC2* exon26 in mature mRNA of KYSE510 cells transfected with siRNAs (siDAZ1-1 and siDAZ1-2). **(D)** A 2706bp DNA fragment including human *TSC2* exons 25, 26 and 27 as well as introns 25 and 26 was cloned into the pcDNA3.1 vector (pcDNA3.1-TSC2-minigene). **(E)** Different alternative splicing patterns of *TSC2* pre-mRNA were detected using RT-PCR in KYSE450 and KYSE510 cells after silencing DAZAP1 or over-expressed DAZAP1.

Accumulated evidence indicates that DAZAP1 plays a crucial function in RNA alternative splicing 35-39. To determine whether DAZAP1-mediated RNA alternative splicing may regulate autophagy of ESCC cells, we performed RNAseq of KYSE510 cells transfected with siDAZ1-1, siDAZ1-2 or NC RNA to identify endogenous splicing events that are controlled by DAZAP1 in ESCC. We observed a large number of significantly changed alternative splicing events upon RNAi of DAZAP1 in ESCC cells. Multivariate analyses of transcript splicing indicated that dysregulated DAZAP1 may lead to alternative splicing of six hundred and thirteen genes, including exon skipping (*n* = 352), intron retention (*n* = 22), alternative 5' splice site (*n* = 34), alternative 3' splice site (*n* = 36), and mutually exclusive exon (*n* = 102) (Figure 5A). The most abundant type of alternative splicing events affected by DAZAP1 was exon skipping which is consistent to previous reports 37,39.

There are eleven alternative splicing genes involved in autophagy control (Figure 5B). Among these genes, *TSC2* (*TSC complex subunit 2*, also known as *tuberin*) codes a GTPase-activating protein (GAP), which acts as a prominent intrinsic regulator of mTORC1 (the mammalian target of rapamycin complex 1) and autophagy processes 40,41. As shown in Figure 5C, silencing DAZAP1 leads to the exclusion of exon 26 to produce a short *TSC2* mRNA isoform compared with the wild type long *TSC2* mRNA. To further verify the splicing patterns of DAZAP1 on the *TSC2* mRNA, we took advantage of a minigene splicing construct containing exon 25, intron 25, exon 26 (an alternative splicing exon), intron 26, and exon 27 of human *TSC2* and transfected it into in KYSE450 and KYSE510 cells (Figure 5D and 5E). RT-PCR with T7 and BGH primers was performed to eliminate endogenous *TSC2* mRNA signals (Figure 5D). The inclusion of the alternative splicing exon (exon 26) was then examined in ESCC cells, either co-transfected with the TSC2-minigene construct, pcDNA3.1-DAZAP1 or different DAZAP1 siRNAs, respectively (Figure 5E and Figure S5). We found that the over-expressed DAZAP1 could promote inclusion of exon 26 in the *TSC2* mRNA (Figure 5E, left panel). In contrast, an obviously elevated exon 26 skipping (the short *TSC2* mRNA isoform) was observed in DAZAP1 knockdown ESCC cells (Figure 5E, right panel).

### DAZAP1 inhibits oncogenic autophagy via the TSC2/RHEB/mTOR signaling

**Figure 6:**
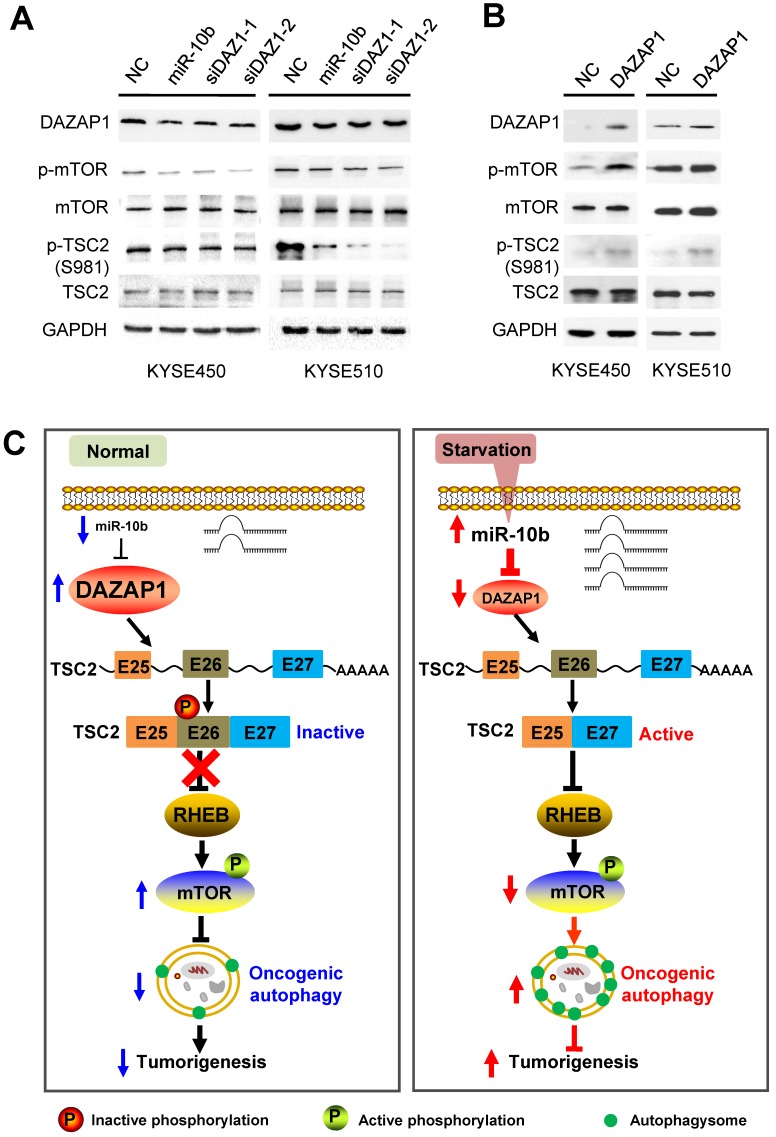
** DAZAP1 inhibits oncogenic autophagy via the TSC2/RHEB/mTOR signal pathway. (A)** KYSE450 and KYSE510 cells were transfected with NC RNA, miR-10b mimics, or *DAZAP1* siRNAs (siDAZ1-1 and siDAZ1-2). After 48h, cell lysates were analyzed by western blotting. GAPDH levels were measured as loading controls. **(B)** KYSE450 and KYSE510 cells were transfected with pcDNA3.1 or pcDNA-DAZAP1. After 48h, cell lysates were analyzed by western blotting. GAPDH levels were measured as loading controls. **(C)** Model for regulation of alternative splicing of *TSC2* mRNA and oncogenic autophagy by DAZAP1 under conditions of nutrient sufficiency.

We further examined how mTOR activities can be controlled through phosphorylation of long and short TSC2 isoforms, and determined the downstream signaling pathway of DAZAP1 in oncogenic autophagy. *TSC2* exon 26 codes the peptide fragment from Leu947 to Arg988 and has a Ser981 residue that can be phosphorylated by AKT 42. The AKT-mediated Ser981 phosphorylation is crucial for translocation of TSC2 from the membrane to the cytosol. Localization of TSC2 to the membrane accounts for the ability of TSC2 to inhibit mTOR signaling via its GAP activity for RHEB (Ras homolog, mTORC1 binding) 42,43. We found that silencing DAZAP1 or expression of an ectopic miR-10b could significantly inhibit phosphorylation of TSC2 Ser981 in exon 26 but did not change TCS2 protein expression in KYSE450 and KYSE510 cells (Figure 6A and Figure S6A). Conversely, over-expressed DAZAP1 could promote phosphorylation of TSC2 Ser981 (Figure 6B and Figure S6B), strongly suggesting that DAZAP1-controlled exon 26 alternative splicing may be critical for TSC2 inactivation in ESCC. The growth factor-responsive TSC2 activation is a major pathway implicated in the negative mTOR regulation 41-43. It is noteworthy that DAZAP1 RNAi or miR-10b mimics dramatically suppressed phosphorylation of endogenous mTOR, but did not impact the protein levels of mTOR (Figure 6A), indicating that the upregulation of endogenous mTOR phosphorylation is not due to the increase of mTOR protein levels. The levels of phosphorylated mTOR significantly increased in ESCC cells with over-expression of DAZAP1 (Figure 6B), which is congruent with the previous notion that activated TSC2 reduces the small GAP activity of RHEB and, thus, inhibits mTOR as well as oncogenic autophagy 41,43. Collectively, our findings demonstrated that starvation-induced DAZAP1 suppression promotes mTORC1-regulated oncogenic autophagy via controlling TSC2 alternative splicing in ESCC (Figure 6C).

## Discussion

Cancer cells up-regulate autophagy and depend on it for survival, growth, and malignancy 14,15,44. However, it is largely unclear how alternative splicing events are involved in nutrient deficiency-induced autophagy of ESCC. In this study, we found that starvation-induced miR-10b promotes oncogenic autophagy of ESCC cells by inhibiting expression of DAZAP1 which is strongly involved in TSC2 RNA splicing. Previous studies demonstrate that AKT inhibits TSC2 functions by phosphorylating its Ser981 residue in exon 26. Consistent with this notion, we observed significantly suppressed phosphorylation of TSC2 Ser981, decreased mTOR phosphorylation and enhanced autophagy in DAZAP1-repressed cells. This study presents an integrated model in which miR-10b activated by environmental cues can suppress DAZAP1 to induce alternative splicing and the activation of TSC2, which promotes autophagy, proliferation and invasion of ESCC cells by regulating the mTOR signaling.

Higher eukaryotes develop one creative way to increase proteome diversity via alternative splicing of pre-mRNA in gene expression 45. In general, alternative splicing is regulated by several splicing regulatory elements (SREs) that recruit trans-acting splicing factors to promote or inhibit splicing 35-39. Multiple splicing factors recognize these SREs in pre-mRNA and compose a functional module to control RNA splicing. From previous studies, DAZAP1 has been seen to be one of these critical splicing factors 35-39. DAZAP1 can regulate splicing of alternative exons through specifically recognizing cis-acting SREs in alternative exons or nearby introns, or indirectly recruiting pre-mRNA targets through binding to the hnRNP A1 family members. Studies on pathological splicing mutations support the importance for DAZAP1 in RNA splicing of several key tumor suppressors, such as *NF1*, *BRCA1* and *ATM* 38. We also observed the dysregulated DAZAP1 leads to alternative exon 26 splicing of tumor suppressor *TSC2* pre-mRNA in ESCC cells.

The role of the TSC2-mTOR signaling in regulating oncogenic autophagy is crucial 40-43. Tumor suppressor TSC2 is a negative regulator upstream of mTOR and its inactivating mutations cause tuberous sclerosis complex, an autosomal dominant syndrome which results in tumor development in multiple organs. TSC2, together with TSC1 and TBC1D7, form the TSC protein complex that functions as a GAP toward the GTPase RHEB and inhibits mTORC1 40-43. Consistently, we found that exclusion of TSC2 exon 26 could avoid its inactivation by Ser981 phosphorylation, leading to the inactivation of the mTOR signaling during starvation-induced autophagy of ESCC cells.

Genetically engineered mouse models have been extensively used in exploring the role of autophagy in cancers and indicated that many aggressive cancers require autophagy for growth, survival, and malignancy 44. In these mouse models, essential autophagy genes, such as *ATG5*, *ATG7*, *ATG13* or *ULK1*, are deleted in genome of tumor cells that arise spontaneously in the context of a normal tumor microenvironment and functional immune system. For example, deletion of *Atg5* or *Atg7* in* Pten^-/-^*-driven prostate cancer, *Kras^G12D^*- or *Braf^V600E^*-driven lung cancer, or *Kras^G12D^*-driven pancreatic ductal adenocarcinoma, suppresses tumor growth. Similarly, deletion of *Atg13* or *Ulk1* in a *Kras^G12D^*-driven glioblastoma decreases tumor progression. As a result, a mouse model with tumor-specific deletion of *miR-10b* in tumors arising spontaneously in mice would be a good tool to stablish the *in vivo* role of the miR10b/DAZAP1/TSC2 axis in ESCC in the future.

In summary, we identified a novel model that integrates splicing control into oncogenic autophagy regulation in ESCC. Environmental conditions can trigger alternative mRNA splicing and autophagy, adding a new layer of gene expression regulation for cancer cells, enabling them to survive in a nutrient-scarce and stressful microenvironment. Ultimately, such insights may enable the rational targeting of the RNA splicing control to unlock the therapeutic potential of ESCC in the clinic.

## Methods

### Cell culture and reagents

Human ESCC KYSE450 or KYSE510 cells were cultured in RPMI 1640 medium (Corning, 31615001) supplemented with 10% fetal bovine serum (FBS; Gibco, 1347575) at 37°C in a 5% CO_2_ incubator. miR-10b mimics, miR-10b inhibitors and small interfering RNA (siRNA) duplexes (siDAZ1-1 and siDAZ1-2) were products of Genepharma (Shanghai, China) (Table S1). The negative control RNA duplex (NC) for miRNA mimics, miRNA inhibitors or siRNAs (Genepharma, Shanghai, China) was nonhomologous to any human genome sequence. All small RNAs were transfected with the INTERFERin reagent (Polyplus, 409-10) as reported previously 46,47. All expression or reporter gene plasmids were transfected with the jetPRIME reagent (Polyplus, 114-07). The mTOR activator MHY1485 was purchased from MedChemExpression Co. (HY-B0795).

### Quantitative reverse transcription PCR (RT-qPCR)

Total RNA was isolated from culture cells or tissue specimens with Trizol reagent (Invitrogen, 94402). To remove genomic DNA, each RNA sample was treated with DNase I (RNase-free) (Thermo Fisher,18068015). Each RNA sample was then reverse transcribed into cDNAs using PrimeScript^TM^ RT Master Mix (TaKaRa, RR036A). Human miRNAs and U6 were detected with their specific stem-loop RT-PCR primers (Ribobio, Guangzhou, China) 47,48. The mRNA expression of *DAZAP1* and other genes were examined with their specific RT-qPCR primers (Table S1).

### Western blot

ESCC KYSE450 and KYSE510 cells were firstly transfected with 20nmol/L miR-10b mimics, miR-10b inhibitor, siDAZ1-1, siDAZ1-2 or NC RNA. Total cellular proteins of KYSE450 and KYSE510 cells were harvested at 48h after transfection. Cell lysates were immunoblotted as previously reported 47,48. Antibodies against LC3B (Cell Signaling, #3868), SQSTM1/p62 (Cell Signaling, #5114),) DAZAP1 (Abcam, ab182558), mTOR (CST, 2983s), p-mTOR (CST, 5536s), TSC2 (Sigma, SAB4503037), p-TSC2(Ser981) (Affinity, AF8330) and GAPDH (Abcam, ab9485) were used. We quantified Western Blot bands with ImageJ.

### GFP-LC3 analyses

After seeding in 12-well plates with a 20mm diameter microscope cover glass (NEST, 801008) in each well, KYSE450 or KYSE510 were transfected with miR-10b mimics, NC RNA, pcDNA-DAZAP1 or the pcDNA3.1 vector, respectively. At 48 h after transfection, KYSE450 or KYSE510 cells were firstly incubated with rabbit monoclonal antibody anti-LC3B (CST, #3868) at 4°C for 12h. ESCC cells were then washed with PBS for 3 times and incubated with Alexa Fluor488-conjugated anti-rabbit IgG (H+L) (CST, #4412) for 1 h at room temperature in the dark. The cells were then washed with PBS for 3 times. Nuclear nuclei were stained with fluoroshield mounting medium with DAPI (Abcam, ab104139) for 3 min in the dark and then washed with PBS for 3 times. The fluorescence of LC3B was observed under the confocal Laser Scanning microscope (ZEISS, LSM 800 with Airyscan, Germany). The average number of FITC-LC3B puncta per cell was counted in three random fields.

### Cell proliferation analyses

A total of 1×10^5^ KYSE450 and KYSE510 cells were seeded in 12-well plates and transfected with small RNAs (20 nmol/L miR-10b mimics, miR-10b inhibitors or NC RNA) or plasmids (the pcDNA3.1 vector or pcDNA-DAZAP1), respectively. Cells were then harvested by trypsin digestion, washed by cold PBS twice, dyed with trypan blue and counted under microscopy.

### Colony formation assays

KYSE450 or KYSE510 (4000 cells per well) were seeded into a 6-well cell culture plate and transfected with various small RNAs (20 nmol/L miR-10b mimics, miR-10b inhibitors, siDAZAP1-1, siDAZAP1-2 or NC RNA) or plasmids (the pcDNA3.1 vector or pcDNA-DAZAP1), respectively. When colonies were visible after 14 days, cells were washed with cold PBS twice and fixed with the fixation fluid (methanol:acetic acid = 3:1). After cells were dyed with crystal violet, the colony number in each well was counted.

### Target gene prediction for miR-10b

It has been reported that intersecting the results of several bioinformatics prediction programs can increase specificity of target gene prediction at the cost of lower sensitivity 49,50. As a result, we chose to integrate the results of four prediction algorithms: TargetScan (http://www.targetscan.org/vert-71/), PICTAR (https://pictar.mdc-berlin.de/), Micro-RNA (http://www.microrna.org) and MiRDB (http://mirdb.org/miRDB/). Overall, nine candidate target genes of miR-10b including *DAZAP1* were found by all algorithms.

### DAZAP1 reporter gene constructs

The sequence corresponding to the wild-type *DAZAP1* 3'-UTR (1445-2105nt) was amplified with KYSE450 cDNA using Pyrobest^TM^ DNA Polymerase (TaKaRa) (PCR primers shown in Table S1). The PCR products with blunt ends were ligated into the appropriately digested pGL3-Control. The resultant plasmid, designated pGL3-DAZAP1, was sequenced to confirm the orientation and integrity. The *DAZAP1* reporter gene plasmid with mutant miR-10b binding site was constructed with QuikChange Site-Directed Mutagenesis kit (Stratagene, La Jolla, CA). These mutant plasmids were confirmed by DNA sequencing and named as pGL3-Mut10b.

### Dual luciferase reporter assays

Both reporter constructs (pGL3-Control, pGL3-DAZAP1, pGL3-Mut10b) plus 20 nmol/L small RNAs (miR-10b mimics or NC RNA) were transfected into KYSE450 and KYSE510 cells. pRL-SV40 (1 ng) (Promega) containing renilla reniformis luciferase was cotransfected to standardize transfection efficiency. Luciferase activities were detected at 48h after transfection using a luciferase assay system (Promega). For each luciferase construct, three independent transfections were done (each in triplicate). Fold increase was calculated by defining the activity of pGL3-Control as 1.

### Wound healing and transwell assays

For wound healing assays, a wound was scratched by a 10μl pipette tip when the cell layer of KYSE450 and KYSE510 reached ~90% confluence. After ESCC cells were continued cultured at 37°C, the average extent of wound closure was quantified. In transwell assays, the transwell chambers were coated with 60 μL BD Biosciences Matrigel (1:20 dilution) overnight in a cell incubator. KYSE450 and KYSE510 cells transfected with various small RNAs or plasmids were added to upper transwell chambers (pore 8 mm, Corning). A medium containing 10% FBS (650 μL) was added to the lower wells. After 48 h, ESCC cells migrated to the lower wells through pores were stained with 0.2% crystal violet solution and counted.

### Patients and tissue specimens

There were eighty-six ESCC patients recruited in the current study. All patients received curative resection for ESCC in Huaian No. 2 Hospital (*n* = 37, Huaian, Jiangsu Province, China) and Shandong Cancer Hospital and Institute (*n* = 49, Jinan, Shandong Province, China) between February 2011 and December 2018. Prior to the surgery, no patients received any local or systemic anticancer treatments. This study was approved by the Institutional Review Boards of Huaian No. 2 Hospital and Shandong Cancer Hospital and Institute. At recruitment, written informed consent was obtained from each subject.

### RNAseq and alternative splicing analyses

To gain insight into the DAZAP1 signaling in ESCC cells, we performed RNAseq of KYSE510 cells trasfected with 20 nmol/L NC RNA, siDAZAP1-1 or siDAZAP1-2 using Illumina Hiseq^TM^ 2500 platform (Illumina). We focused on identification of differential alternative splicing events since DAZAP1 is a key regulator of alternative splicing 35-39. We utilized multivariate analysis of transcript splicing 51 to detect differential alternative splicing events between NC RNA and siDAZ1-1 or NC RNA and siDAZ1-2. Various kind of alternative splicing forms (exon skipping, intron retention, alternative 5' splice site, alternative 3' splice site, and mutually exclusive exon) were examined. The RNAseq data have been deposited at the National Center for Biotechnology Institute Gene Expression Omnibus (GEO) repository under accession number GSE134376.

### miniGene constructs and *in vitro* splicing assays

To validate the alternative splicing of the *TSC2* pre-mRNA, we firstly amplified the genomic sequence of human *TSC2* spanning from exon 25 to exon 27 using the genomic DNA of KYSE510 as the template (PCR primers shown in Table S1) 39,52,53. The PCR products were cloned into the appropriately digested pcDNA3.1. The resultant plasmid was named as pcDNA3.1-TSC2-minigene. For the splicing analyses, we co-transfected pcDNA3.1-TSC2-minigene with various small RNAs (siDAZ1-1, siDAZ1-2 or NC RNA) or plasmids (the pcDNA3.1 vector or pcDNA-DAZAP1), respectively, into KYSE450 or KYSE510 cells. Total RNA of each sample was extracted at 48 h after transfection. Expression of splicing variations derived from the minigene was detected through RT-PCR (primers shown in Table S1).

### Statistics

The difference between two groups was calculated using Student's *t* test (assuming Gaussian distributions) or Wilcoxon Signed Rank Test (not assuming Gaussian distributions). Impacts of miR-10b and DAZAP1 expression on ESCC patients' survival was tested by Kaplan-Meier plots. A *P* value of less than 0.05 was used as the criterion of statistical significance. All analyses were performed with SPSS software package (Version 16.0, SPSS Inc.) or GraphPad Prism (Version 5, GraphPad Software, Inc.).
