# Supplementary material for: Starvation-induced suppression of DAZAP1 by miR-10b integrates splicing control into TSC2-regulated oncogenic autophagy in esophageal squamous cell carcinoma
Source: Theranostics. 2020 Apr 6;10(11):4983–96. doi: 10.7150/thno.43046 (PMC7163442; doi:10.7150/thno.43046)

Table S1. Oligonucleotides used in the current study

#	**Oligonucleotide Sequences (5'→3')**
DAZAP1 3'-UTR reporter gene constructs	
DAZAP1-3'-UTR-F	CCGCTCGAGGAGGGCAGAACCACAACG (XhoI)
DAZAP1-3'-UTR-R	CTAGAAGCTTACTACCAAGCAGGTCAGTCG (HindIII)
Mutagenesis 3'-UTR-F	GTCACTGGTTCAACAGAGTTAGCTAAAAAAATGTCTTCA
Mutagenesis 3'-UTR-R	TGAAGACATTTTTTTAGCTAACTCTGTTGAACCAGTGAC
TSC2 minigene constructs	
TSC2-minigene-F	CGGGGTACCGGCCTGCGGTCCAATGTCCTCTTGTC (KpnI)
TSC2-minigene-R	CCGCTCGAGCTCTTCGGGACAGCCGTGAAGTTGGAG (XhoI)
TSC2 minigene alternative splicing PCR primers	
T7-minigene-F	TAATACGACTCACTATAGGG
BGH-minigene-R	TAGAAGGCACAGTCGAGG
DAZAP1 siRNA duplexes	
siDAZAP1-1	CCCAGGAGCGAUAACAGUATT/UACUGUUAUCGCUCCUGGGTT
siDAZAP1-2	GAGACUCUGCGCAGCUACUTT/AGUAGCUGCGCAGAGUCUCTT
DAZAP1 qRT-PCR primers	
DAZAP1-qF	GCGGTCTTGACTGGAGCA
DAZAP1-qR	TTCGGCCATCTAGCGTGT
TFAP2C qRT-PCR primers	
TFAP2C-qF	CTGTTGCTGCACGATCAGACA
TFAP2C-qR	CTCAGTGGGGTTCATTACGGC
RAP2A qRT-PCR primers	
RAP2A-qF	ATGCGCGAGTACAAAGTGGT
RAP2A-qR	GCGACGAATCCACCTCGAT
NCOR2 qRT-PCR primers	
NCOR2-qF	TGCAGATCATCTACGACGAGA
NCOR2-qR	TCCGCATCGCCTGGTTTATTT
MDGA2 qRT-PCR primers	
MDGA2-qF	CTTTGACCATACCTGCCATCAC
MDGA2-qR	ATTTTCACCTCACGGCCAATC
GTF2H1 qRT-PCR primers	
GTF2H1-qF	TGCAGGGGACACAACTAACTT
GTF2H1-qR	ACTTGACTCACAACAAGGTCTTT

DOCK11 qRT-PCR primers	
DOCK11-qF	CTTGGGCCAAATTGGAGACAA
DOCK11-qR	CCCACAGCATTTCACGGAC
CSMD1 qRT-PCR primers	
CSMD1-qF	TGGAGGAGATTCCAGTCGCT
CSMD1-qR	GCATAGTTCGGATACCCGTGA
β-actin qRT-PCR primers	
β-actin-qF	GGCGGCACCACCATGTACCCT
β-actin-qR	AGGGGCCGGACTCGTCATACT

Supplementary Figure 1

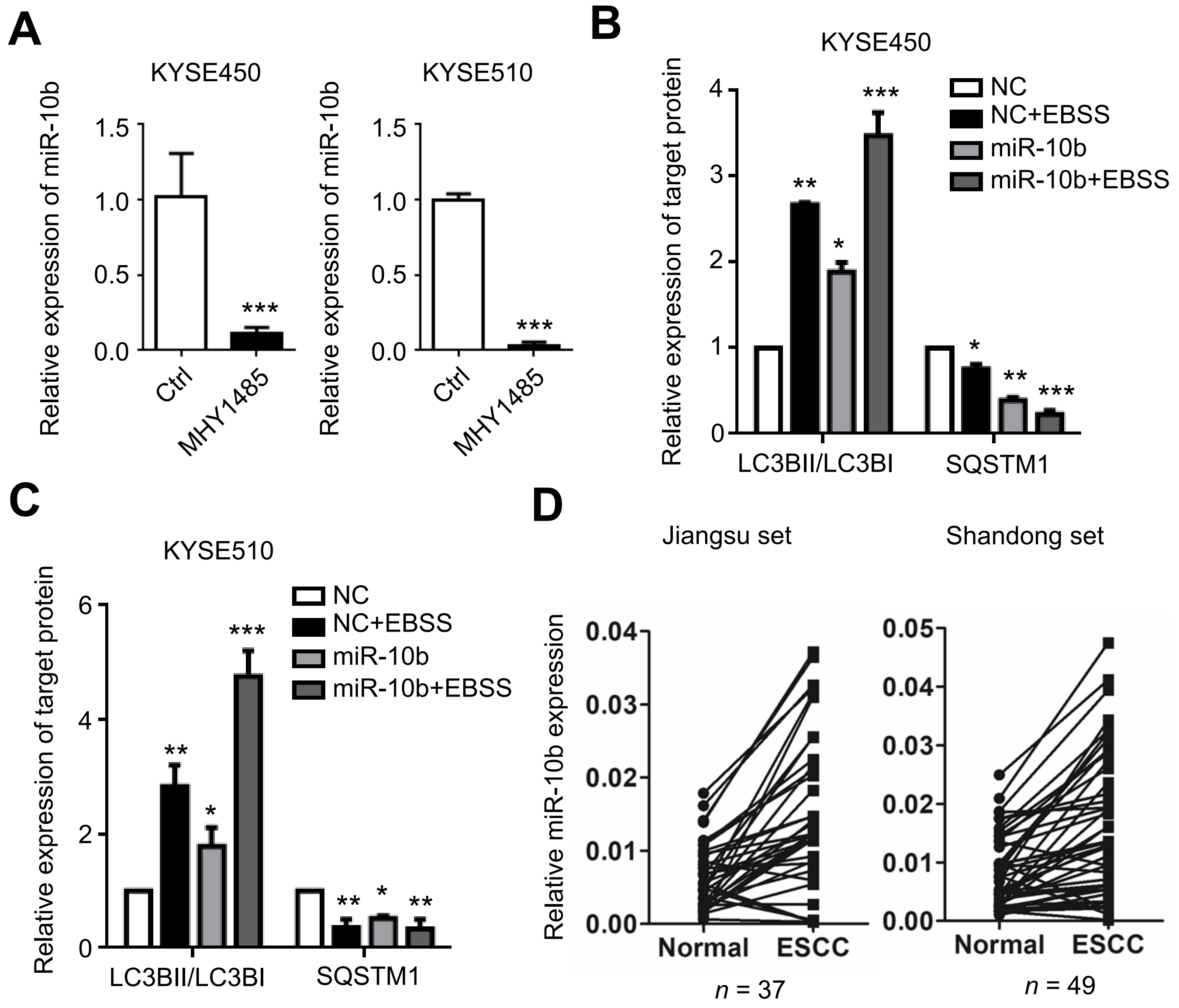

Supplementary Figure 2

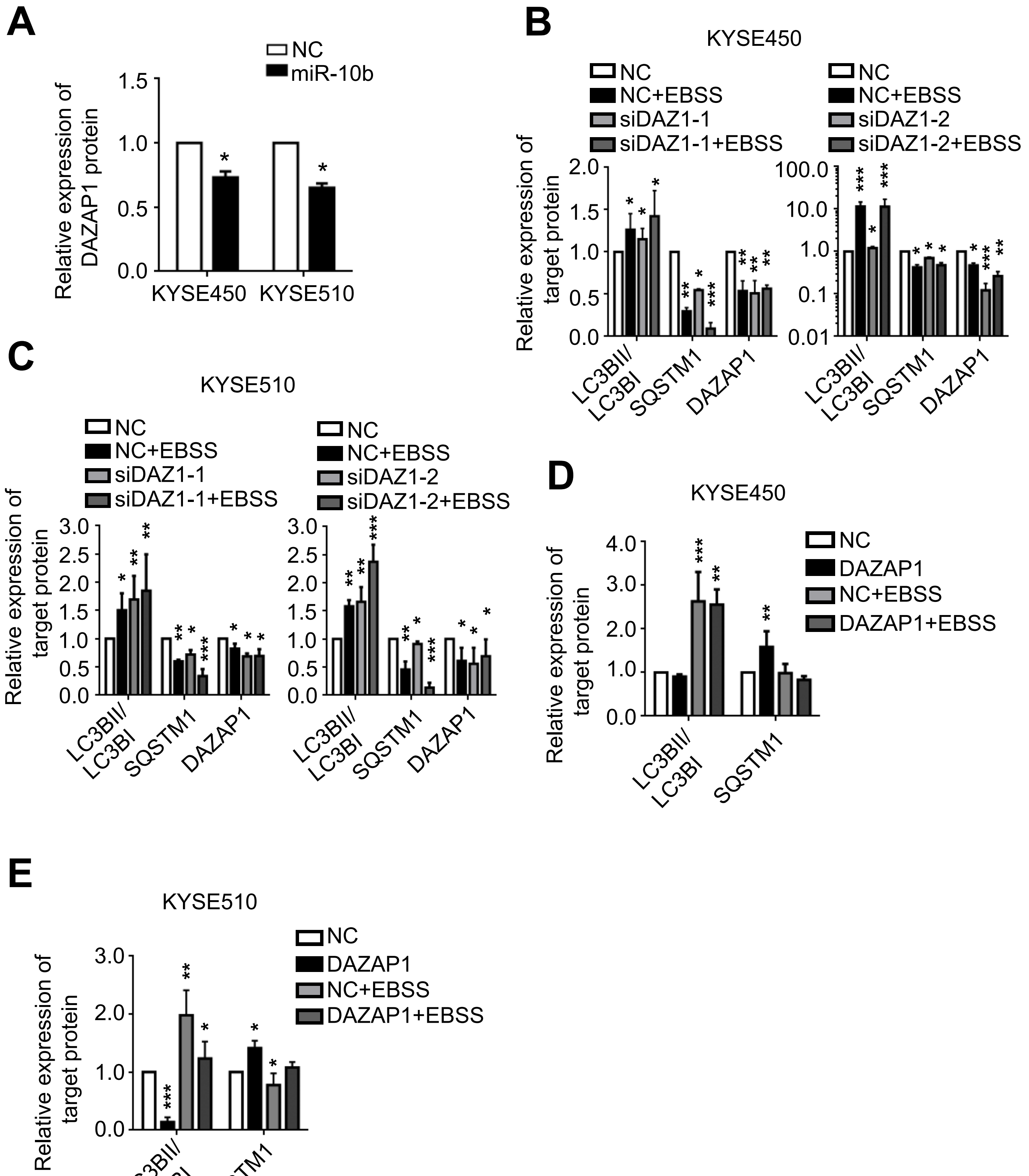

Supplementary Figure 3

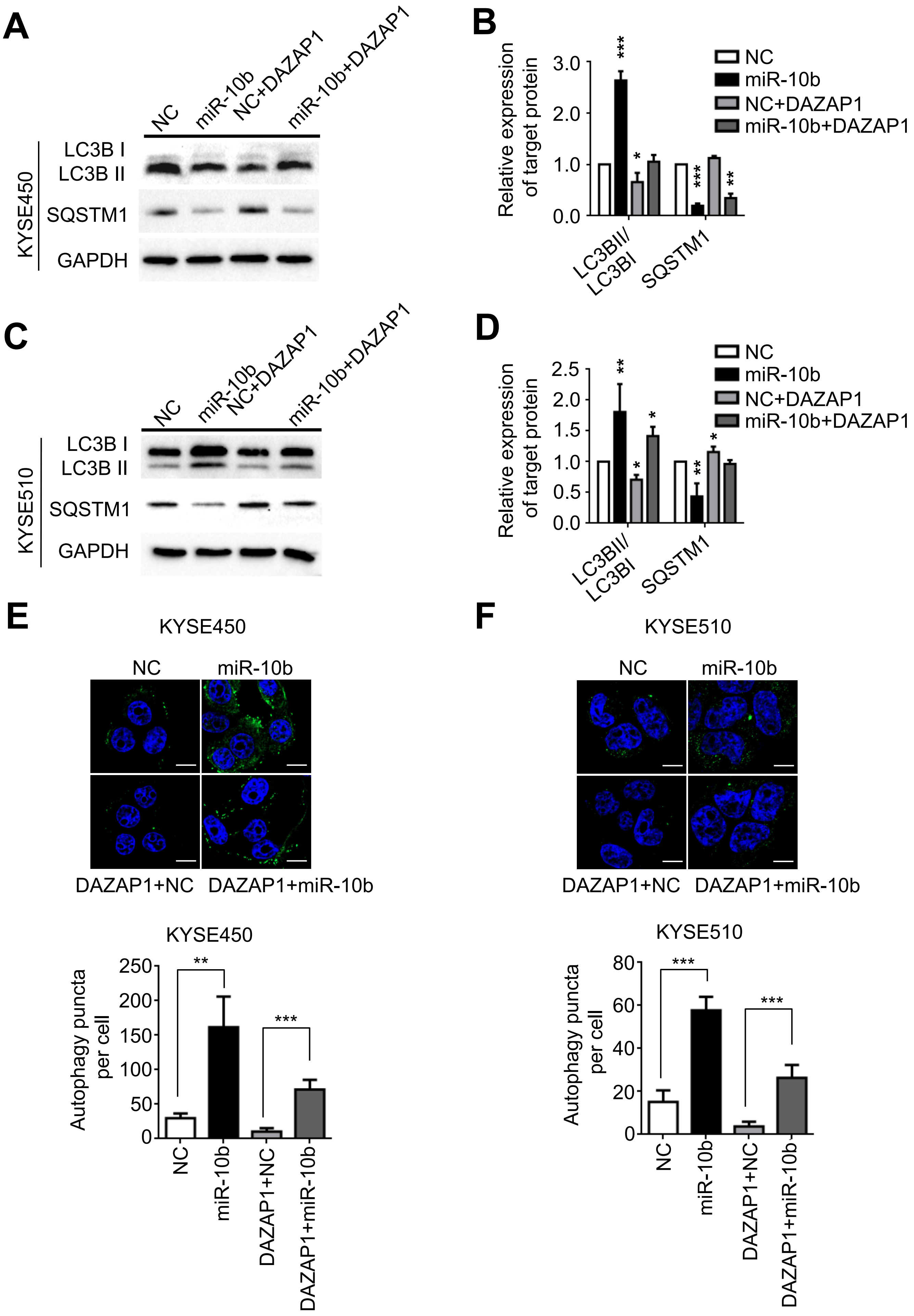

Supplementary Figure 4

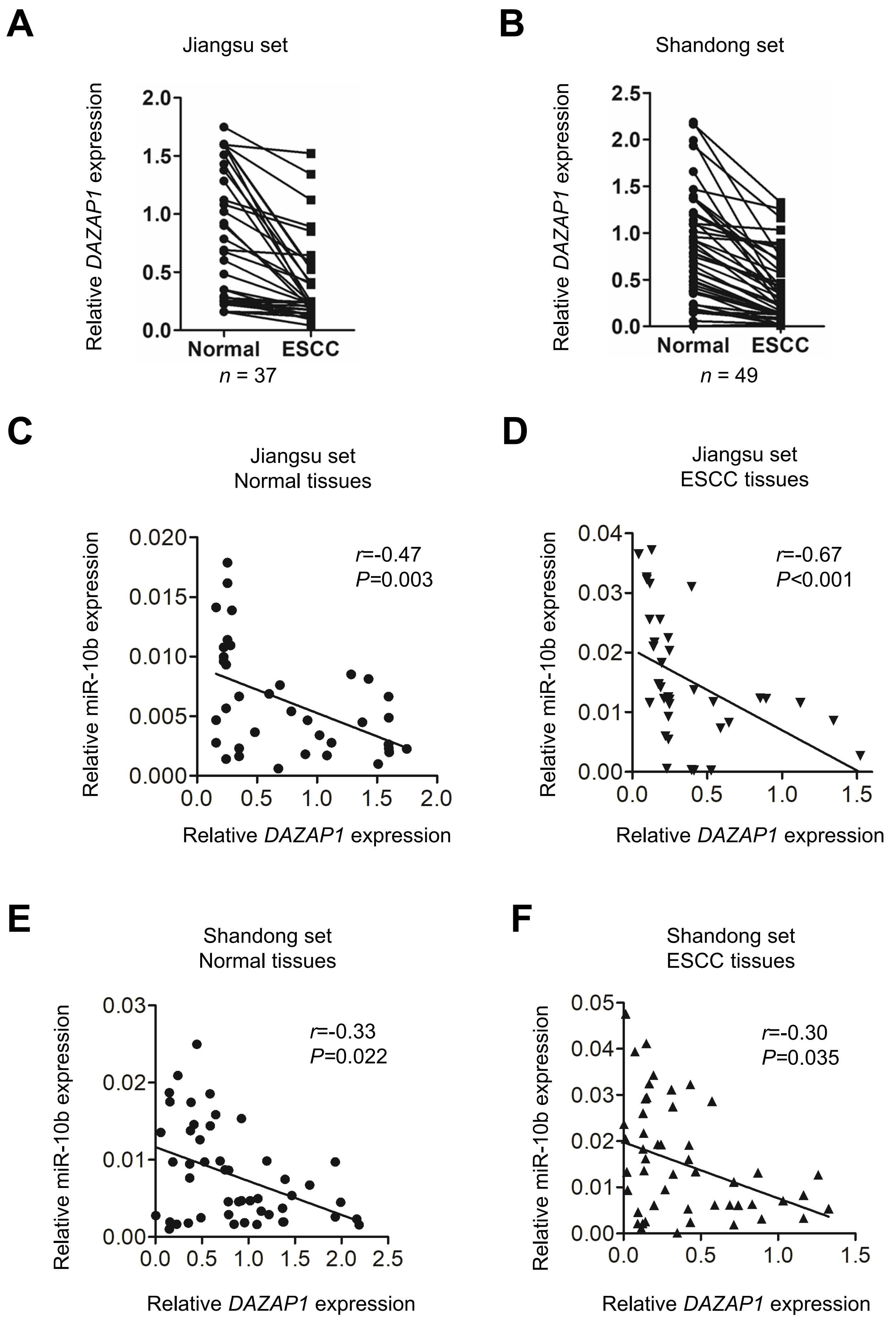

Supplementary Figure 5

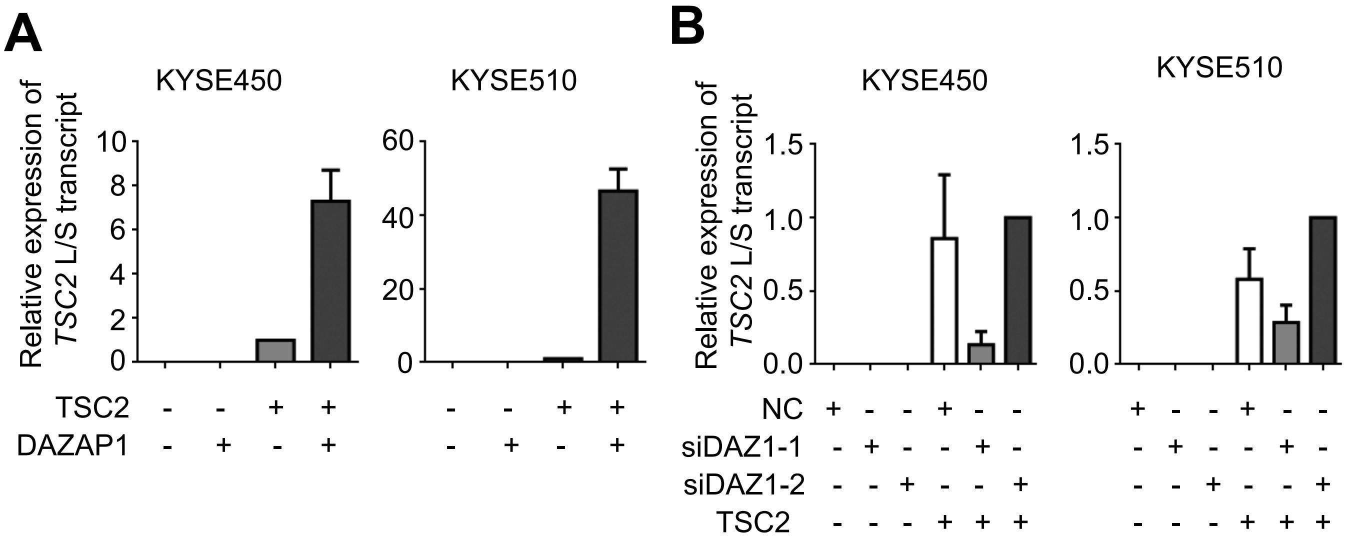

Supplementary Figure 6

A

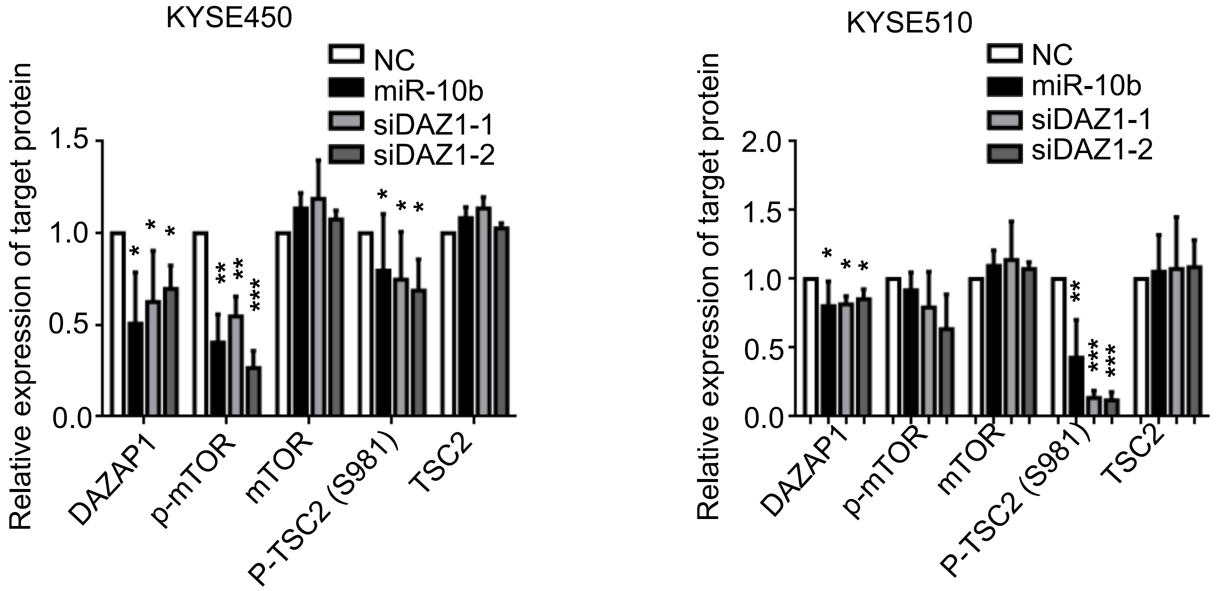

B

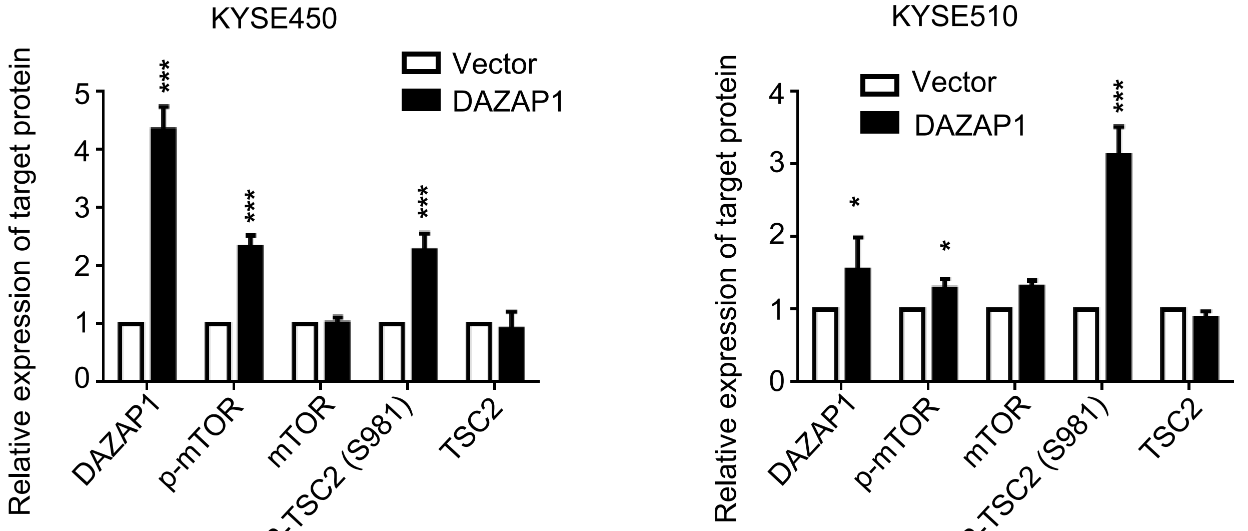

Supplement: Supplementary file 1 — Supplementary figures and tables. [file thnov10p4983s1.pdf]
